# Revisiting fungal polarized growth: from Spitzenkörper to crescent accumulation of secretory vesicles

**DOI:** 10.1016/j.isci.2025.114067

**Published:** 2025-11-15

**Authors:** Adrien Hamandjian, Glen Calvar, Matthieu Blandenet, Mélanie Crumière, Nathalie Poussereau, Mathias Choquer, Christophe Bruel

**Affiliations:** 1 Université Claude Bernard Lyon 1, CNRS, INSA-Lyon, UMR5240 Microbiologie, Adaptation et Pathogénie, 10 Rue Raphaël Dubois, 69622 Villeurbanne, France; 2 Bayer SAS, Crop Science Division, Laboratoire Mixte, 14 Impasse Pierre Baizet, 69263 Lyon, France

**Keywords:** Microbiology, Cell biology

## Abstract

Polarized elongation in filamentous fungi depends on secretory vesicles being supplied and fusing at the apex. In Ascomycota and Basidiomycota, these vesicles have mostly been reported to accumulate into a spheroidal structure known as the Spitzenkörper. Using time-lapse microscopy, the spatial and temporal dynamics of fluorescently labelled vesicles were investigated in 13 apices of the phytopathogenic fungus *Botrytis cinerea*. Time-projections of the fluorescent signal highlighted the spheroid Spitzenkörper in half the sample and a crescent-shaped region of the apical dome in the other half. A linear relationship was found between the roundness of the fluorescent region and the hyphal elongation rate. Temporal dynamics clustering and Fourier transform showed periodic pulses of fluorescence intensity in hyphae displaying a Spitzenkörper that were not detected in those exhibiting a crescent-shaped accumulation. These results reveal a dual mode of secretory vesicle accumulation at the apex of growing hyphae from the same culture.

## Highlights

• Apical vesicle accumulation can be spatially stable (Spitzenkörper) or more dynamic
• Distinct modes of apical vesicle organization coexist and sustain hyphal elongation
• Elongation rate is higher in hyphae maintaining a Spitzenkörper over time
• Periodic events of vesicle accumulation and release occur at the Spitzenkörper

## Graphical abstract

**Figure:**
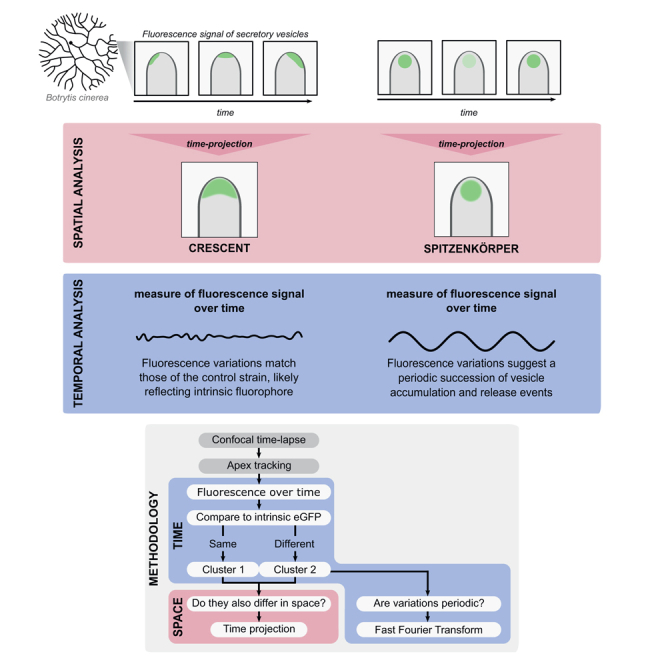

## Introduction

Sustained polar cell elongation is a shared trait in various branches of the tree of life, from filamentous bacteria such as actinomycetes,^1^ to tip-growing eukaryotic cells found in plants^2^^,^^3^^,^^4^ (e.g., pollen tubes and root hairs), fungi, oomycetes and algae^5^ (e.g., protonemata). In eukaryotic microorganisms, it has been described in filamentous yeasts, including pathogens such as *Candida albicans*^6^^,^^7^ or *Ashbya gossypii*^8^ whose respective virulence to human and cotton relies on the production of filaments. More notably, polar cell elongation is a hallmark of filamentous fungi, supporting continuous hyphal extension.

Polarized cell elongation results from the spatial restriction of exocytosis within the fungal cell. By limiting vesicle fusion to a specific region, lipids are locally supplied to the plasma membrane, resulting in the unidirectional elongation of the cell.^9^ The development of the mycelium, formed by extending and branching hyphae, therefore depends on the production, transport, and regulated exocytosis of secretory vesicles (SV) at the hyphal tip. SV are Golgi-derived vesicles transported to the apex by microtubules^10^^,^^11^^,^^12^ or directly produced on-site by apical Golgi compartments.^13^ Once at the hyphal tip, also called the apex, SV are locally transported along actin filaments to interact with the exocyst, a protein complex that facilitates vesicle docking and fusion with the plasma membrane during exocytosis.^14^^,^^15^^,^^16^

In addition to transporting and releasing lipids, SV also carry transmembrane proteins destined for the plasma membrane, and soluble proteins delivered to the extracellular space, including the cell wall. Among the transmembrane protein cargoes, chitin synthases (CHS) have received particular attention. CHS are type 2 glycosyltransferases that synthesize chitin, a major structural component of the fungal cell wall that is composed of N-acetylglucosamine residues linked by β-(1,4) linkages.^17^ Because CHS are transported by SV to the apical plasma membrane, fluorescently tagged CHS have been widely used to visualize SV trafficking.^11^^,^^18^^,^^19^^,^^20^ The delivery by SV of enzymes involved in cell wall synthesis and remodeling ensures that cell wall expansion occurs in coordination with plasma membrane extension during apical elongation.

SV are the final carriers of all proteins that follow the canonical secretory pathway, i.e., proteins translated in the endoplasmic reticulum and matured in the Golgi apparatus.^21^ These proteins include lytic enzymes involved in fungal nutrition, which play a key role in ecosystems due to their importance in nutrient cycling.^22^^,^^23^ Additionally, SV transport proteins that mediate interactions between fungi and other organisms, notably virulence factors secreted by pathogenic species.^24^ Beyond its ecological function, fungal secretion is also crucial to various industrial processes, ranging from fermentation in the food industry to the secretion of proteins and enzymes of economic interest.^25^ Consequently, a deeper understanding of temporal and spatial dynamics of exocytosis in fungi is needed to better understand their biology, but also to better control pathogens or, on the contrary, maximize production yields in industrial settings.

In Ascomycota and Basidiomycota vesicles can accumulate at the apex of elongating filaments to form a spheroidal structure known as the Spitzenkörper.^26^ This structure has been proposed to act as a vesicle supply center, where vesicles accumulate before being delivered to the plasma membrane.^27^ The Spitzenkörper is located at the interface between the microtubules transporting SV to the apex,^28^ the polarisome nucleating actin filaments at the apex,^29^ and the exocyst mediating the vesicle tethering and fusion with the plasma membrane.^30^ In *Neurospora crassa*, it was shown to be stratified in two SV subpopulations known as microvesicles and macrovesicles accumulating at the core and in the outer layer of the Spitzenkörper, respectively.^31^ Besides its pivotal position in the exocytic flux, the Spitzenkörper is also tangled with endocytosis. In *Aspergillus nidulans,* the class III chitin synthase ChsB exemplifies this connection. After delivery to the plasma membrane by exocytosis, it is retrieved by endocytosis at the endocytic collar, recycled through the trans-Golgi network, and redirected to the Spitzenkörper for exocytosis.^32^ This continuous cycle of secretion and recycling emphasizes the role of the Spitzenkörper as a central hub of vesicle trafficking at the apex.

The Spitzenkörper can therefore be described as both dynamic and stable. It is dynamic by the constant entry and exit of vesicles,^26^^,^^33^^,^^34^^,^^35^ but stable by its importance to direct growth in actively growing filaments.^36^^,^^37^^,^^38^^,^^39^ This stability is, however, challenged. In the filament-producing yeast *A. gossypii*, chemical labeling of lipids showed the presence of a Spitzenkörper in fast elongating hyphae, but a crescent-shaped accumulation of vesicles in slower elongating hyphae.^40^ Interestingly, both Spitzenkörper and crescent-shaped accumulation of SV have also been documented in *A. nidulans*.^19^^,^^41^ While significant progress has been made in understanding the apical machinery and vesicle trafficking during polarized cell elongation, the consistency of vesicle accumulation through time and space across filaments remains unclear. In addition, studies on polarized cell elongation have mainly focused on cells displaying a Spitzenkörper and have been conducted in saprotrophic model species, potentially underestimating the diversity of vesicle accumulation patterns and behaviors, both between hyphae and across fungal species.

In this study, a fluorescently labelled class III chitin synthase was used to label SV in the phytopathogenic necrotrophic fungus *Botrytis cinerea*. Time-projections of time-lapse confocal acquisitions revealed two populations of growing hyphae: one showing a spheroidal accumulation of fluorescence at the apex, consistent with a Spitzenkörper, and the other showing a crescent-shaped accumulation. Further temporal analysis revealed periodic variations of fluorescence intensity at the apex of hyphae displaying a Spitzenkörper, which were not detected in hyphae displaying a crescent. These results reveal the existence of at least two populations of hyphae that display different spatial and temporal dynamics of SV distribution in the apical dome.

## Results

### The fluorescently labelled class III chitin synthase BcCHSIIIa localizes at the tip of growing hyphae in *B. cinerea*

To visualize SV at the apex of *B. cinerea* hyphae, a reporter system was required. Based on the successful labeling of SV through the fluorescent tagging of class III chitin synthases in different Ascomycetes,^11^^,^^18^^,^^19^^,^^20^^,^^41^ the *B. cinerea* chitin synthase IIIa (BcCHSIIIa - Bcin04g03120) was chosen.^42^ The gene encoding an optimized version of the fluorescent protein eGFP^43^ was fused at the 3′ end of the BcCHSIIIa gene by homologous recombination to produce the chimeric BcCHSIIIa::eGFP protein (Figure S1). In parallel, a control strain was constructed that produces eGFP alone under the control of the constitutive pOliC promoter (eGFP^Cyt^ strain).

**Figure 1:**
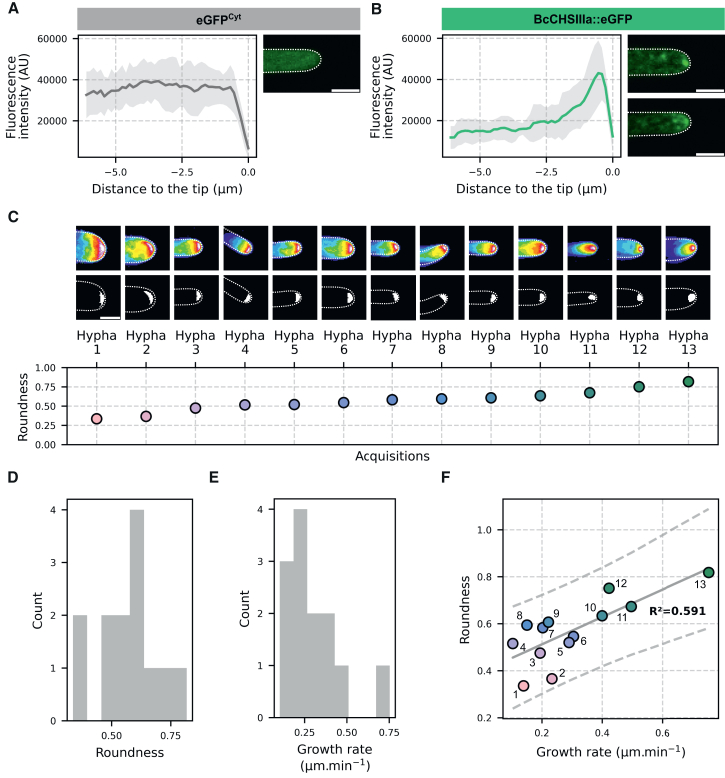
Distinct accumulation patterns of BcCHSIIIa::eGFP correlate with hyphal growth rates (A and B) Fluorescence intensity measured along the longitudinal axis of growing hyphae from the eGFP^Cyt^ strain (A) and the BcCHSIIIa::eGFP strain (B). Solid lines represent the mean fluorescence intensity, shaded areas indicate standard deviation (*n* = 5 for A, *n* = 12 for B). Confocal images show representative hyphae (scale bars = 3 μm). Panels C–F show the BcCHSIIIa::eGFP strain only. (C, top) Time projections obtained for each acquisition after apex tracking, showing the overall fluorescence distribution over the acquisition time. (C, middle) Regions of fluorescence accumulation, defined by the pixels with the top 4.5% of intensity in (C, top). Dotted white lines outline hyphae. Regions of interest displayed in (C) represent regions of 6.78 × 6.78 μm. (C, bottom) Scatterplot shows roundness values measured from regions of accumulation in (C, middle). Point color encodes roundness. Scale bars = 3 μm. (D) The distribution of roundness measures follows a normal distribution (Shapiro-Wilk test, *p*-value 0.965). (E) The distribution of growth rate measures also follows a normal distribution (Shapiro-Wilk test, *p*-value 0.063). (F) Scatterplot of roundness versus growth rate for each acquisition (*n* = 13). The solid gray line shows the ordinary least-squares fit. The dashed gray lines indicate the 95% prediction interval for individual observations. Points are color-coded by roundness as in (C). Numbers refer to Hyphae 1–13 as defined in (C).

Following growth on a glass surface, confocal microscopy was performed on both strains. As expected, the eGFP^Cyt^ control strain displayed a diffuse cytosolic fluorescent signal (Figure 1A). In contrast, mobile fluorescent speckles and an apical accumulation of fluorescence were observed in the BcCHSIIIa::eGFP strain (Figure 1B) that were consistent with previously reported localizations of SV transporting chitin synthases.^18^^,^^19^^,^^20^^,^^41^ These observations, therefore, validated the creation of the desired reporter strain.

### Patterns of apical BcCHSIIIa::eGFP accumulation are linked to hyphal elongation rate

Noticeably, in different hyphae of the BcCHSIIIa::eGFP strain, the fluorescence signal at the apex appeared either as a spheroid spot or was more diffused (Figure 1B, right panel top and bottom). To exclude the possibility of an artifact, 10 min time-lapse acquisitions (1 frame every 4.455 s) were collected on 13 BcCHSIIIa::eGFP hyphae. For each acquisition, the apex was tracked and cropped to account for hyphal elongation. The overall distribution of fluorescence over the course of the acquisition was visualized by performing time-projections (Figure 1C, top). The region of maximal fluorescence accumulation was extracted for each hypha, using a threshold corresponding to the top 4.5% brightest pixels (Figure 1C, middle). This threshold value consistently yielded regions larger than 0.5 μm^2^, allowing reliable roundness measurements, while remaining restricted to the region of fluorescence accumulation (Figure S2). In some hyphae, this region of maximal fluorescence appeared as a spheroid below the plasma membrane, indicating that the BcCHSIIIa::eGFP-transporting vesicles remained spatially confined throughout the acquisition in these hyphae. In other hyphae, this region appeared as a crescent. In the corresponding acquisitions, the fluorescence in each individual frame was located below the plasma membrane and illuminated either the whole apical cap, sections of it, or even submembranous spots. Upon time projection of all the individual frames, this dynamic localization of the BcCHSIIIa::eGFP vesicles over time formed the observed crescent. To quantitatively compare the spheroid and crescent patterns between hyphae, we measured the area and roundness of the fluorescence accumulation region, and ordered the hyphae according to their roundness values (Figure 1C, bottom). The area of fluorescence accumulation had a mean of 0.806 ± 0.155 μm^2^ and the roundness values followed a normal distribution (Shapiro-Wilk test, *p*-value 0.965), ranging from 0.336 to 0.818 (mean roundness of 0.571 ± 0.131). These observations revealed that the apical region of fluorescence accumulation spans a continuum of shapes, from crescent-like (lower roundness) to spherical (higher roundness).

For each acquisition, kymographs were then generated to estimate the growth rate of each hypha during acquisition (Figure S3). These rates ranged from 0.104 to 0.753 μm min^−1^ (mean value 0.301 ± 0.173 μm min^−1^), with no statistical difference observed between the control (eGFP^Cyt^) and the BcCHIIIa::eGFP strain (Student’s *t* test, *p*-value 0.191, mean growth rates 0.450 ± 0.247 μm min^−1^ and 0.301 ± 0.173 μm min^−1^ with *n* = 5 and *n* = 13, respectively). In contrast, a positive linear correlation was observed between the roundness of the apical fluorescent region and the hyphal elongation rate in the BcCHSIIIa::eGFP strain (Figure 1F, Pearson correlation r = 0.769, *p*-value 0.002, *n* = 13, analysis of residuals in Figure S4).

### Temporal dynamics clustering reveals two clusters based on fluorescence variability over time

In addition to analyzing the spatial organization of SV at the apex, we examined the temporal dynamics of their accumulation. For each frame of all acquisitions, the mean intracellular fluorescence intensity was measured in the apical region (4 μm from the tip, see STAR Methods) of hyphae from the control (eGFP^Cyt^) and BcCHSIIIa::eGFP strains. This yielded, for each acquisition, a time series representing the mean fluorescence intensity at the apex over time. In all time series, this intensity fluctuated over time, and control z-stack imaging indicated that these fluctuations arose independently of movements in the z dimension (Figure S5). Since such fluctuations can result from intrinsic properties of eGFP (folding, maturation, or stability), we compared the dynamics observed in the two strains.

**Figure 2:**
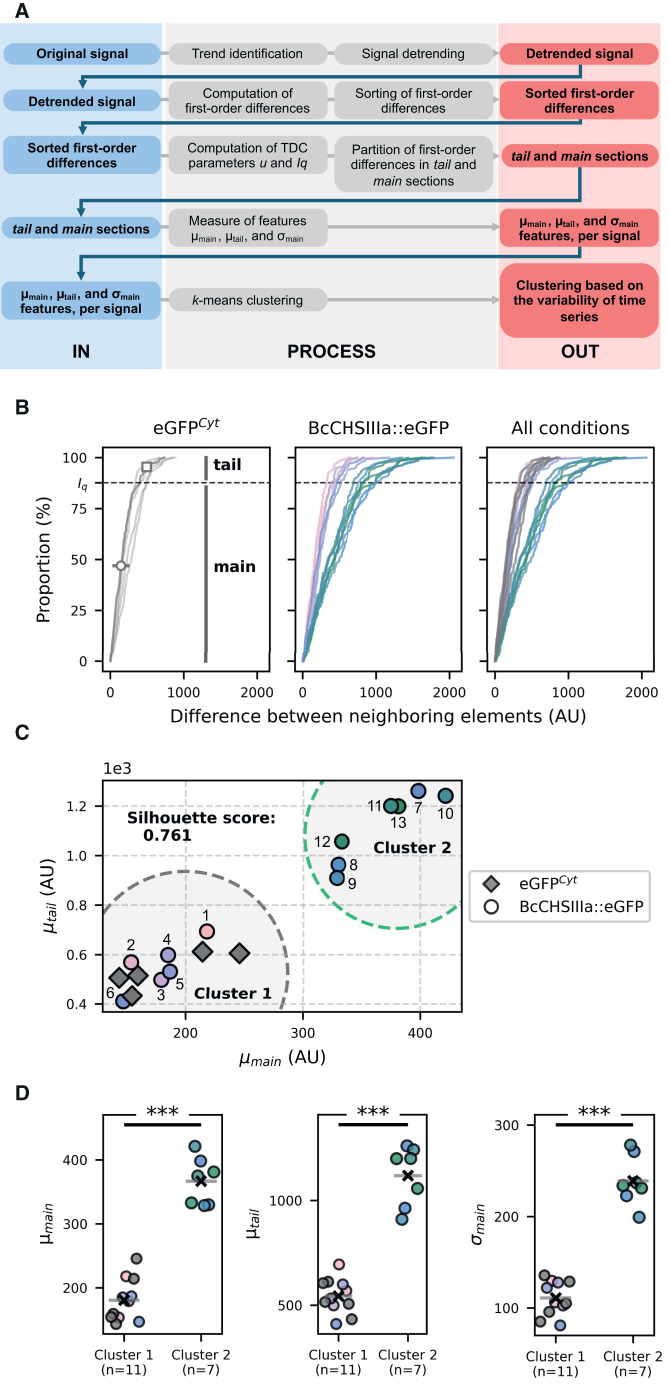
Temporal dynamics clustering reveals two distinct clusters differing in their variability over time (A) Workflow of the temporal dynamics clustering (TDC) approach. Signals are detrended, absolute first-order differences are computed, sorted, and shown as cumulative distributions. Using the TDC parameters *u* and *I_q_*, each distribution is split into *main* and *tail* sections. For each acquisition, we extract three features: the mean of elements in the *main* section (μ_main_), the standard deviation of elements in the *main* section (σ_main_), and the mean of elements in the *tail* section (μ_tail_). These features are then used in *k*-means clustering to group signals by fluorescence variability. (B) Sorted first-order differences for the control eGFP^Cyt^ strain (left, *n* = 5), the BcCHSIIIa::eGFP strain (middle, *n* = 13), and the combined dataset (right). Each curve corresponds to one signal. The dashed horizontal line marks *I_q_*, the threshold separating the *main* and *tail* sections. For one eGFP^Cyt^ example, μ_main_ is shown as a circle, σ_main_ as a horizontal line centered on μ_main_, and μ_tail_ as a square. (C) Scatterplot of μ_tail_ versus μ_main_ for all acquisitions, with eGFP^Cyt^ shown as diamonds (*n* = 5) and BcCHSIIIa::eGFP as circles (*n* = 13). Dashed outlines mark the approximate *k*-means cluster contours for visual guidance only (not decision boundaries). (D) Comparison of μ_main_, μ_tail_, and σ_main_ between Cluster 1 and Cluster 2 (Student’s *t* test *p*-values: 6.7e-9, 7.7e-9, and 2.7e-9). For each Cluster, the cross and horizontal line indicate the position of the mean. In plots, (∗∗∗) indicates a *p*-value < 0.001.

To characterize the temporal dynamics of the fluorescence signals, we applied Temporal Dynamics Clustering (TDC),^44^ a features-based clustering approach that groups time series based on fluorescence variability over time. As shown in Figure 2A, the signals were first detrended, and the absolute first-order differences were then computed for each time series, before being sorted and displayed as cumulative distributions (Figure 2B) that capture the variability of the signal over time. After processing, each confocal acquisition therefore yielded one cumulative distribution which could then be divided into two sections using the TDC parameters *u* and *I_q_*: the *main* section, representing common variations, and the *tail* section, capturing extreme fluctuations, respectively (Figure 2B). From these sections, three features were extracted: the mean of elements in the main section (μ_main_) and its associated standard deviation (σ_main_), and the mean of elements in the tail section (μ_tail_). As an example, a signal with these features highlighted is shown in Figure 2B. Thus, each acquisition could ultimately be represented by the three features μ_main_, μ_tail_, and σ_main_. These features were then used for *k*-means clustering, which identified two distinct clusters, termed Cluster 1 and Cluster 2 (Figure 2C).

The clustering quality was supported by a silhouette score of 0.761, indicating strong and well-defined separation between clusters. Moreover, statistical analysis revealed significant differences between the two clusters for all 3 TDC metrics (Figure 2D, Student’s *t* test, *p*-values of 6.7e-9 for μ_main_, 2.7e-9 for σ_main_, and 7.7e-9 for μ_tail_). These differences in the 3 metrics suggest that fluorescence variability between clusters differs not only in extreme events (μ_tail_) but also in their overall temporal behavior (μ_main_). In contrast, the median fluorescence intensity before detrending was not significantly different between clusters (Mann-Whitney U test, *p-*value 0.073), suggesting that the distinction arose from differences in temporal variability rather than absolute intensity. Together, these results demonstrate the presence of two statistically distinct patterns of apical fluorescence dynamics in the 13 hyphae sample.

### Distinct fluorescence dynamics are linked to the roundness of apical vesicle accumulation

Cluster 1 included all the acquisitions recorded on the 5 hyphae from the control eGFP^Cyt^ strain. Since clustering was based on fluorescence variability over time, Cluster 1 therefore, likely reflects the intrinsic fluorescence fluctuations of eGFP itself. Interestingly, the 13 acquisitions recorded on the 13 hyphae from the BcCHSIIIa::eGFP strain were distributed across both clusters (6 in Cluster 1 and 7 in Cluster 2, Figure 2C). This indicated the presence of two populations of growing hyphae in the BcCHSIIIa::eGFP strain, differing in apical fluorescence variability over time. In all the hyphae grouped in Cluster 2, the temporal fluorescence behavior at the apex was distinct from the one observed in hyphae of the control strain, suggesting a specific dynamic of SV in these hyphae.

**Figure 3:**
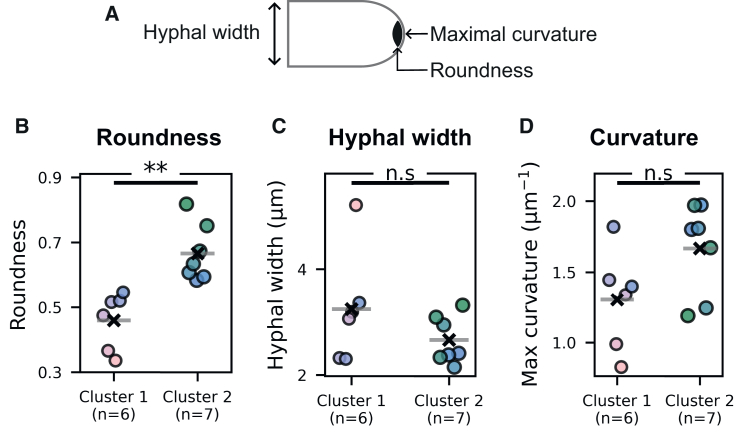
Roundness of the apical fluorescence region differs between TDC clusters (A) Illustrative diagram shows hyphal width, the point of maximal curvature, and the apical fluorescence-accumulation region used to measure roundness. (B) Roundness measured from time-projection images of BcCHSIIIa::eGFP using the top 4.5% mask (Figure 1C), grouped by TDC clusters (*n* = 6 for Cluster 1 and *n* = 7 for Cluster 2, Student’s *t* test *p*-value 0.001). (C) Hyphal width grouped by cluster (Student’s *t* test, *p*-value 0.214). (D) Maximum apex curvature grouped by cluster (Student’s *t* test, *p*-value 0.081). (B–D) Points are colored by roundness as in Figures 1 and 2. For each Cluster, the cross and horizontal line indicate the position of the mean. In plots, (ns) denotes non-significant tests (*p-*value > 0.05), (∗) indicates *p-*value < 0.05, (∗∗) indicates *p-*value < 0.01, and (∗∗∗) indicates *p-*value < 0.001.

Interestingly, a link between clustering and the roundness of the apical fluorescent signal was noticed. Cluster 1 and Cluster 2 differed in the roundness of the region of apical fluorescence accumulation over time, with mean values of 0.460 ± 0.080 and 0.666 ± 0.082, respectively (Student’s *t* test, *p*-value 0.001, Figure 3B). To verify that no single acquisition accounted for the effect, we performed a leave-one-out Student’s *t* test, removing individual acquisitions in turn. The assumptions of normality and homoscedasticity were met in all iterations, and the difference remained significant throughout (largest *p*-value 0.007). This confirmed that the observed difference was robust and not driven by any single acquisition. Hyphae in Cluster 1 displayed lower roundness values than those in Cluster 2, with no overlap between the two groups. In contrast, hyphae in Cluster 1 and 2 did not significantly differ in width or apex curvature (Figures 3C and 3D, Student’s *t* test *p*-value 0.214, Student’s *t* test *p*-value 0.08, respectively). This indicates that the temporal dynamics that separate the acquisitions of Cluster 1 from those of Cluster 2 are associated with differences in the spatial organization of the SV-related apical fluorescence. On one hand, the hyphae belonging to Cluster 1 displayed an accumulation of fluorescence at the apex with low roundness values, and, over time, variations in fluorescence intensity could not be distinguished from the intrinsic fluorescence variations displayed by cytosolic eGFP. On the other hand, the hyphae belonging to Cluster 2 displayed an accumulation of fluorescence at the apex with higher roundness values. In these hyphae, variations in fluorescence intensity over time statistically differed from the intrinsic dynamics of eGFP, suggesting significant fluctuations in vesicle accumulation. Because roundness values span from crescent-like (low roundness) to spherical (high roundness), the fluorescence accumulation in hyphae from Cluster 1 will hereafter be referred to as crescent-shaped, while it will be referred to a Spitzenkörper in hyphae from Cluster 2.

### Fluorescence variations are periodic in hyphae exhibiting a Spitzenkörper

**Figure 4:**
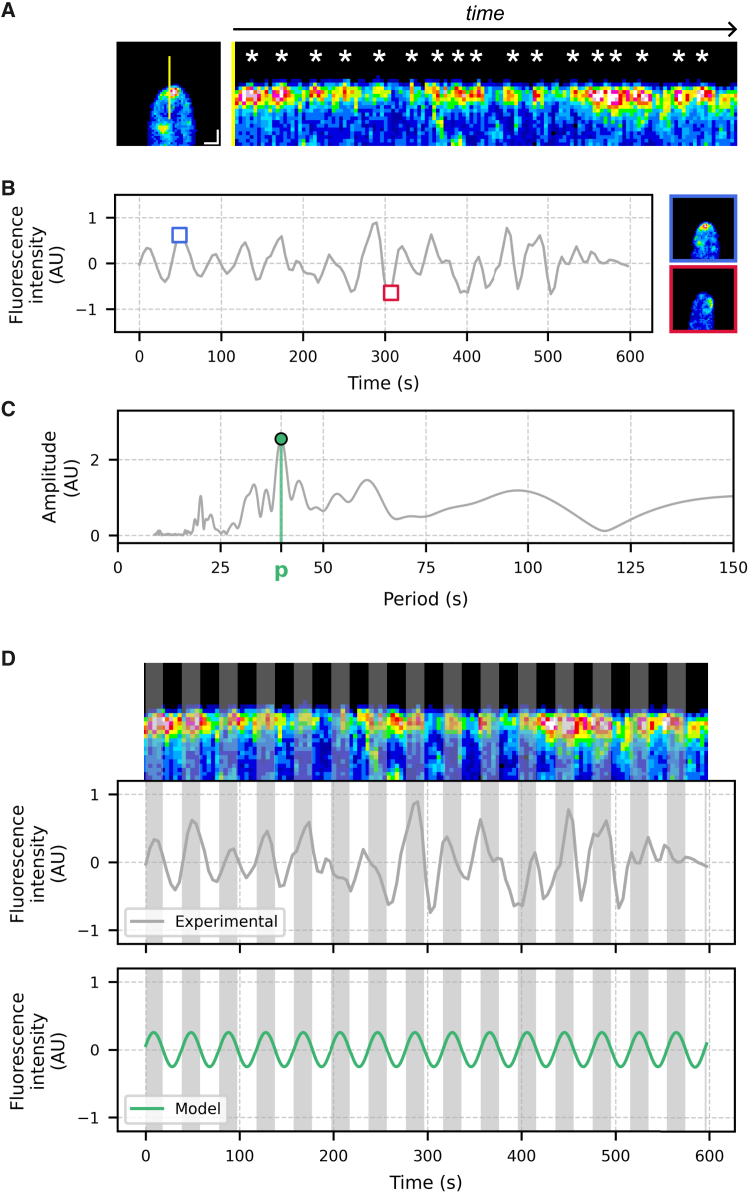
The spherical accumulation of fluorescence is marked by periodic variations of fluorescence (A, left) Frame at t_0_ after tracking and cropping of the apex in the acquisition used as an example (Hypha 12). The yellow line indicates the position used to generate the kymograph. Scale bars = 1 μm. (A, right) Kymograph of the Hypha 12 acquisition showing fluorescence intensity along the 3.8 μm line (yellow line in the left panel, y axis of the kymograph) over 135 frames (10 min in total, 4.455 s interval, *x* axis of the kymograph). The absence of slope results from the dynamic tracking of the apex. White asterisks mark the approximate positions of fluorescence hotspots. (B) Mean fluorescence intensity per frame over time obtained for Hypha 12. The blue square highlights a time point of high fluorescence and its corresponding frame (top right). The red square marks a low-intensity time point and its associated frame (bottom right). (C) Amplitude spectrum from fast Fourier transform analysis of the time series in (B), restricted to periods below 150 s. The green marker indicates a peak corresponding to a period of 39.75 s. (D, top) Kymograph and time series (middle) corresponding to hypha 12 and a sinusoidal model with a period of 39.75 s (bottom). Gray zones represent predicted fluorescence peaks from the sinusoidal model and are overlaid on both the time series and the kymograph.

The production of kymographs from the time-lapse acquisitions of the BcCHSIIIa::eGFP strain revealed occurrences of fluorescence hotspots, as exemplified in Figure 4A for one acquisition and in Figure S3 for the others. These spots reflected spatially and temporally restricted events of fluorescence accumulation, which appeared as oscillations when the mean fluorescence intensity at the apex was plotted against time (Figure 4B). Noticeably, these events appeared regularly spaced, suggesting the presence of an underlying periodic behavior. Control z-stack imaging indicated that these fluorescence fluctuations arose from apical accumulation dynamics rather than the displacement of the signal along the z axis (Figure S5).

**Figure 5:**
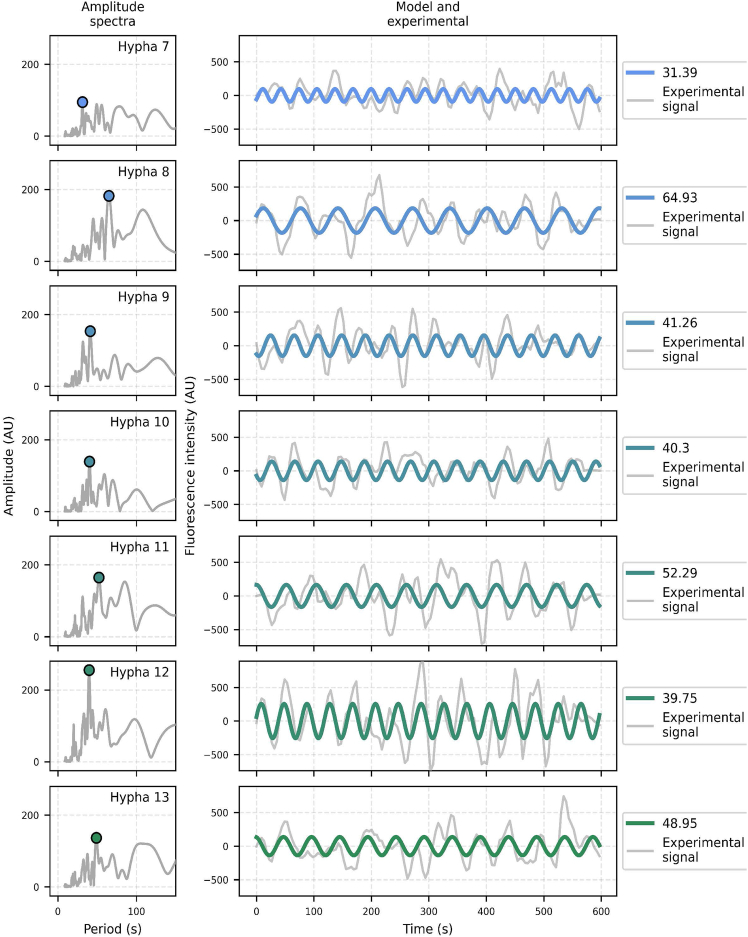
All hyphae assigned to Cluster 2 display a dominant oscillatory component (Left) Amplitude spectra for acquisitions belonging to TDC Cluster 2. Colored markers indicate the peak of maximal amplitude in each spectrum. For each acquisition, the peak amplitude and the corresponding period were used to generate a sinusoidal model (bold colored line), overlaid on the experimental signal (gray line). Acquisitions are ordered by the roundness of the fluorescence accumulation region, from lowest (top) to highest (bottom). Periods indicated in the figure are expressed in seconds. Sinusoidal models are color-coded according to roundness, consistent with previous figures.

To evaluate the presence of a putative period governing the detected oscillations in acquisitions of Cluster 2, the Fast Fourier Transform (FFT) was used. Pre-processed time series were analyzed (see STAR Methods) and the outputs were expressed as amplitudes over periods, producing amplitude spectra. In the example shown in Figure 4C, the amplitude spectrum revealed a prominent oscillatory component with a period of 39.75 s. When this period was used to generate a sinusoidal model (Figure 4D, green line), this model closely aligned with the experimental data (Figure 4D, gray line). This indicated that the identified period adequately captured the fluorescence variations observed at the apex. Comparable results were obtained across all acquisitions grouped in Cluster 2 (Figure 5), each displaying a dominant oscillator with periods ranging from 31.39 to 64.93 s (mean 45.55 ± 10.09 s, *n* = 7), and this estimate was robust to the exclusion of any single acquisition. Short-time Fourier transform analysis further showed that, despite local fluctuations, the dominant period identified for each acquisition was largely maintained across the acquisition time (Figure S6). The relationship between oscillation period and hyphal elongation rate was finally examined by Pearson correlation (Figure S7). In the full dataset, no correlation was detected (Pearson correlation 0.033, *p*-value 0.944). A leave-one-out analysis showed that Hypha 8 had a major influence and that excluding it yielded a positive trend that approached but did not reach significance (Pearson correlation 0.747, *p*-value 0.088). We observed a similar pattern for the relationship between oscillation period and roundness (Figure S7). Taken together, these results suggest that such relationships may exist, yet the current sample size and the influence of a single acquisition prevent a definitive conclusion. Future studies, including larger datasets will be required to validate or invalidate these putative correlations between oscillation and elongation rate or roundness.

## Discussion

Polar cell elongation is a hallmark of vegetative growth in filamentous fungi, and the apical accumulation of secretory vesicles (SV) in a Spitzenkörper has been broadly documented in Ascomycota and Basidiomycota hyphae. Scarce observations of hyphae showing vesicle accumulation in a crescent have nonetheless been reported,^19^^,^^40^^,^^41^ suggesting a possible diversity of SV accumulation patterns in growing hyphae. Here, we used the fusion of a class III chitin synthase (common SV marker) with the fluorescent protein eGFP to study the apical accumulation of SV in growing hyphae of the plant phytopathogen *Botrytis cinerea*. As expected, a concentrated accumulation of fluorescence was observed at the apex of growing hyphae. Interestingly, this accumulation appeared either as a spheroid or as a dynamic submembranous signal in different hyphae of the same culture. The detection of a fluorescent spheroid reflects a spatially restricted accumulation of SV and is consistent with the observation of a Spitzenkörper in *B. cinerea* hyphae by electron microscopy.^45^ As for the detection of a dynamic submembranous accumulation, it reflects a spatially less restricted accumulation of SV at the apex, comforting similar reports in *A. nidulans*^19^ and in the filamentous yeast *A. gossypii*,^40^ and therefore suggesting a possible general characteristic in filamentous fungi.

To better understand the plurality of apical vesicle accumulations in hyphae of the same culture, we tracked several apices over time and generated time-projections to assess how SV were organized at the apex during hyphal elongation. In hyphae with a Spitzenkörper, time-projections produced a spherical structure, consistent with strong spatial control of SV over time. In the other hyphae, the fluorescence localized dynamically across different submembranous regions of the apical cap, in such a way that the time projections yielded crescent-shaped footprints. Roundness measurements revealed that the crescent- and Spitzenkörper-like footprints represent the two ends of a continuous spectrum.

We then investigated whether roundness was correlated with elongation rate, and a positive linear correlation was found. This relationship is reminiscent of findings in *A. gossypii*, where the organization of the polarisome and exocyst, two complexes known to interact with the Spitzenkörper, depends on growth rate, with faster-growing hyphae displaying a spherical organization and slower-growing ones showing a crescent-like arrangement.^40^ Similarly, in *N. crassa*, germlings lacking a Spitzenkörper exhibit lower elongation rates than mature hyphae in which a Spitzenkörper is present.^34^ Altogether, these findings suggest an interdependence between Spitzenkörper formation and hyphal elongation. However, whether vesicle accumulation facilitates faster growth or, conversely, rapid growth promotes Spitzenkörper assembly remains to be elucidated. In addition, we also observed hyphae growing at similar rates (0.233 and 0.221 μm min^−1^, for example) and exhibiting dissimilar apical SV organizations (crescent and Spitzenkörper with roundness of 0.366 and 0.607, respectively, in this example). Intermediate elongation rates may therefore be permissive to either the presence or absence of the Spitzenkörper. Such a buffer zone was observed in *A. gossypii*, where similar elongation rates were measured in hyphae with crescent or Spitzenkörper-like organizations of the exocyst or of the polarisome.^40^ Altogether, this suggests a not-strictly linear relationship between elongation rate and SV organization, and these observations across species could support a model in which SV apical accumulation undergoes growth rate-dependent reorganization, transitioning from a crescent to a Spitzenkörper as the elongation rate increases. With none of our recordings capturing hyphae undergoing a substantial change in elongation rate, such a transition could not be observed. Whether such a reorganization occurs progressively within individual hyphae or whether crescent and Spitzenkörper represent distinct and stable states associated with different hyphal populations, therefore, remains an open question.

We next investigated the temporal dynamics of apical fluorescence accumulation by analyzing the mean fluorescence intensity measured over time from the same time-lapse confocal acquisitions used for time-projections. This analysis revealed clear fluctuations in intensity over time. To characterize these temporal patterns, we applied Temporal Dynamics Clustering^44^ (TDC), which identified two clusters. Cluster 1 grouped time series whose variations were indistinguishable from those of the control strain expressing cytosolic eGFP, whereas Cluster 2 grouped time series with marked fluctuations. Remarkably, Cluster 1 and Cluster 2 also differed significantly in the roundness of the region of apical fluorescence accumulation over time, with all hyphae grouped in Cluster 1 showing a crescent (low roundness) and those grouped in Cluster 2 displaying a Spitzenkörper (high roundness). Differences in the spatial organization of SV accumulation are therefore also associated with differences in temporal behavior (the fluorescence in hyphae displaying a Spitzenkörper exhibits marked variations of intensity over time).

We further characterized the temporal dynamics of Cluster 2 acquisitions using Fourier transform analysis. Cluster 1 acquisitions were not analyzed because their fluorescence fluctuations did not statistically differ from those of the cytosolic control. In all Cluster 2 signals, a dominant oscillatory component was found, indicating a repeated succession of high and low fluorescence intensity events regularly spaced over time. This suggests the existence of periodic successions of SV accumulation and dispersion events at the apex of the Cluster 2 hyphae, as previously described in *N. crassa* and *A. nidulans*,^46^ as well as in *Arthrobotrys flagrans*.^18^ Interestingly, the study by Takeshita et al.^46^ led to the proposition of an inverse relationship between the hyphal elongation rate and the oscillation period across species, linking periodic SV release events to the pulsed hyphal elongation already documented by López-Franco et al*.*^47^ In *N. crassa*, elongation is rapid (12 μm min^−1^) and the period governing the apical fluorescent oscillation is short (17 ± 6 s).^46^ In *A. nidulans*, elongation is slower (1.8 μm min^−1^) and the period is longer (30 ± 7 s).^46^ Our results in *B. cinerea* would support this trend with an even slower hyphal mean elongation rate (0.34 ± 0.21 μm min^−1^) and a longer period governing the apical fluorescent oscillation (45.55 ± 10.09 s). While the methods used to identify this periodicity differ (measurement of the temporal distance between fluorescence peaks in the study of Takeshita et al.^46^ and Fourier transform analysis in our study), the observed pattern suggests that faster elongation rates may be associated with shorter oscillation periods at the species level. Such a relationship could be explained by the demand for lipids at the plasma membrane. Assuming a stable size of SV, an increase in elongation rate would indeed require an increase in lipid supply, made possible by a reduction in the time between exocytic events.

Within our *B. cinerea* dataset, however, we also observed the opposite tendency with a positive, yet non-significant, linear correlation between period and growth rate. One possibility is that, within a species, faster elongation may require longer recovery times between exocytic events, in contrast to the cross-species trend. This longer period could accommodate the re-establishment of polarity known to be diluted by exocytic events^48^^,^^49^ or part of a feedback mechanism with cell wall forces.^50^ Additional acquisitions and comparative studies across fungi will be needed to test whether such intra-species dynamics represent a general principle.

Altogether, our study integrates spatial and temporal analyses that revealed, within the same culture of *B. cinerea*, two subpopulations of growing hyphae that differ in the dynamics of SV accumulation at the apex. One subpopulation (Cluster 2) displays periodic fluctuations reflecting cycles of vesicle accumulation and release, whereas the other (Cluster 1) does not. Spatially, hyphae exhibited either spheroid or crescent footprints of apical fluorescence, connected by a continuum of roundness values. This indicates that the spatial stabilization of the vesicle pool at the apex is a prerequisite for periodic behavior. In other words, once the vesicle pool remains consistently localized, dynamic oscillations of accumulation and release become detectable, whereas below this stabilization threshold, the signal is too dispersed for periodicity to manifest or to be detected. The dichotomy between crescent and Spitzenkörper is therefore indicative of a change in apical organization, and recognizing the plasticity of SV accumulation may reshape current models of fungal growth regulation, with broader implications for fungal development, adaptability, and pathogenicity. Since vesicular traffic and apical structure are closely linked to the cytoskeleton, the transition between crescent-like and Spitzenkörper accumulation may reflect underlying remodeling of actin or microtubule networks. This possibility is supported by recent work documenting actin structures embedded within the Spitzenkörper^51^ and by SV distributing in a crescent-similar way when the myosin V transport is partially inactivated through the ablation of the HUM complex components in *A. nidulans*.^52^ This type V myosin being an effector of Rab11,^52^ one could consider that a modulation of Rab11 in some *B. cinerea* hyphae could inactivate the myosin and lead to the formation of a crescent. A perturbation of the actin skeleton could also impair the myosin-dependent transport of SV and lead to a crescent. However, such perturbations would likely have an impact on the apex morphology or expansion rate, while these parameters were found to be similar in the hyphae exhibiting a crescent or a Spitzenkörper. The molecular basis of the SV accumulation plasticity remains to be explored.

At last, our study establishes a standardized pipeline as a proof of concept for quantifying the temporal behavior and spatial organization of apical vesicle accumulation. This approach can be extended to other fungi and to conditions enabling further exploration of the relationship between apical vesicle dynamics and elongation rate, as well as to the diversity that may exist across species. It also opens the way to studies using mutants or chemical inhibitors to dissect the mechanisms governing apical organization in space and time. Importantly, our findings in *B. cinerea*, together with evidence from other fungi, indicate that apical dynamics are more diverse than often assumed. Future studies on hyphal elongation should therefore acknowledge this diversity, as focusing solely on hyphae with a Spitzenkörper may capture only a subset of fungal behavior and risks overlooking alternative strategies of polarized growth.

### Limitations of the study

While class III chitin synthases (CHS) are commonly used to visualize secretory vesicles via fusion with a fluorescent protein, it is important to note that these enzymes may be transported by only a subpopulation of vesicles. In *N. crassa*, CHS are specifically transported by microvesicles, while the Spitzenkörper also contains macrovesicles.^31^ In *B. cinerea*, transmission electron microscopy has revealed the presence of both micro- and macrovesicles, but the distribution of BcCHSIIIa between these subpopulations remains unknown.^45^

Although the confocal acquisition protocol was standardized, it was not optimized for yield, limiting the number of usable acquisitions. Additional movies would refine precision, notably for correlation analyses. However, the leave-one-out analyses, together with residual and influence diagnostics for the roundness-growth correlation, indicate that our conclusions are not driven by any single acquisition. The present sample size is adequate for the conclusions drawn.

We were also limited by the spatial and temporal resolution of our microscopic acquisitions, which imposed constraints on several analyses. Limited spatial resolution reduces the accuracy of roundness measurements, while the acquisition frame rate restricted the precision of elongation rate estimates and lowered the resolution of Fourier transform-based analyses of temporal dynamics. Such limitations could be overcome in future studies using faster and higher-resolution imaging modalities.

Lastly, the signal pre-processing used in this study was intentionally stringent to minimize false positives, but it may also have filtered out biologically informative signals, notably low-frequency patterns. Future work will therefore be needed to explore temporal dynamics that may have gone undetected here.

## Resource availability

### Lead contact

Requests for further information and resources should be directed to the lead contact, Adrien Hamandjian (contact@adrien-h.com).

### Materials availability

Fungal strains generated and used in this study are available from the lead contact upon request.

### Data and code availability

• Data have been deposited at Zenodo and are publicly available as of the date of publication at https://doi.org/10.5281/zenodo.16419340.
• All original codes have been deposited at Zenodo and are publicly available as of the date of publication at https://doi.org/10.5281/zenodo.16419340.
• Any additional information required to reanalyze the data reported in this article is available from the lead contact upon request.

## Acknowledgments

We thank Isabelle Gonçalves for her critical reading of the article and Amélie de Vallée for her crucial contribution to establishing the Western blot detection of the BcCHSIIIa::eGFP protein. The authors also thank Bayer SAS for access to specific equipment. This work was supported by the Université Claude Bernard Lyon 1, the 10.13039/501100004794CNRS and Bayer SAS.

## Author contributions

A.H. conceived the study, performed the experiments, analyzed the data, developed the software tools, and prepared the figures. G.C., M.B., and M.Cr. produced the fungal strains used in this study. N.P., M.Ch., and C.B. secured funding and supervised the project. AH wrote the initial draft of the article, which was reviewed and edited by A.H., M.Ch., and C.B., with input from all authors.

## Declaration of interests

The authors declare no competing interests.

## STAR★Methods

### Key resources table

**Table:** 

REAGENT or RESOURCE	SOURCE	IDENTIFIER
**Antibodies**		

Anti-GFP from mouse IgG1κ (clones 7.1 and 13.1)	ROCHE	11814460001; RRID: AB_390913
Goat anti-Mouse IgG (H + L) Cross-Adsorbed Secondary Antibody, HRP	INVITROGEN	G-21040; RRID: AB_2536527

**Bacterial and virus strains**		

*Agrobacterium tumefaciens* LBA1126	Rolland et al.^54^	LBA1126

**Chemicals, peptides, and recombinant proteins**		

PDA medium	THERMO SCIENTIFIC	CM0139B
MMII minimal medium	N/A	Composition in STAR Methods
Protease inhibitor cocktail	SIGMA-ALDRICH	P8215
Bolt™ Bis-Tris 4–12% gels	INVITROGEN	NW04125BOX
Bolt™ MES SDS Running buffer	INVITROGEN	B0002
Laemmli buffer	N/A	Composition in STAR Methods
SuperSignal West Femto	THERMO SCIENTIFIC	34094

**Deposited data**		

Raw and pre-processed confocal images	This study	https://doi.org/10.5281/zenodo.16419340
Extracted datasets	This study	https://doi.org/10.5281/zenodo.16419340
Python and FIJI analysis scripts	This study	https://doi.org/10.5281/zenodo.16419340
*Botrytis cinerea* reference genome genome assembly ASM83294v1	EnsemblFungi	https://fungi.ensembl.org/Botrytis_cinerea/Info/Index?db=core

**Experimental models: Organisms/strains**		

*Botrytis cinerea* B05.10 (wild type)	NCBI	NCBI:txid332648
*Botrytis cinerea* BcCHSIIIa::eGFP	This study	N/A
*Botrytis cinerea* eGFP^Cyt^	This study	N/A

**Oligonucleotides**		

AtMT_S-IIIa-GFP-RFG-F (TCAGTTGGTTCAT GTTATCTTCTTTCT)	This study	N/A
AtMT_S_GFP-R (GGACATTGCACGGGATTACTT)	This study	N/A

**Recombinant DNA**		

p7 plasmid	Blandenet et al.^58^	N/A
pBHT2 plasmid	Mullins et al.^60^	N/A

**Software and algorithms**		

FIJI (ImageJ version 1.54p)	Schindelin et al.^61^	https://imagej.net/software/fiji/
Python (version 3.11.5)	Python Software Foundation	https://www.python.org
Pandas (version 2.0.3)	The pandas development team	https://doi.org/10.5281/zenodo.17229934
SciPy (version 1.11.1)	Virtanen et al.^68^	https://scipy.org/
NumPy (version 1.23.5)	Harris et al.^65^	https://numpy.org/
Kappa plugin (version 2.0.0)	Mary and Brouhard^62^	https://doi.org/10.1101/852772
KymographBuilder (version 3.0.0)	Mary et al.^63^	https://imagej.net/plugins/kymograph-builder
Custom Python script and FIJI macro	This study	https://doi.org/10.5281/zenodo.16419340

**Other**		

ZEISS Axio Observer 7	ZEISS	N/A
LSM 800 Laser Scanning Confocal Module	ZEISS	N/A
Plan-Apochromat 63×/1.40 NA Oil DIC objective	ZEISS	420782-9900-799
96-well SensoPlate microplate	GREINER BIO-ONE	655892

### Experimental model and subject details

#### Culture and growth conditions

For all strains, conidia were harvested after 10 days of culture at 21°C with near-UV light on a medium containing glucose 0.5%, malt extract 0.1%, tryptone 0.1%, casamino acids 0.1%, yeast extract 0.1%, ribonucleic acids 0.02% and agar 1.6%.^53^ Conidia were then stored at −80°C and defrosted on ice before use in experiments.

For confocal acquisitions, conidia were diluted to 1 × 10^3^ conidia.mL^−1^ in MMII medium (NaNO_3_ 24 mM, glucose 111 mM, KH_2_PO_4_ 0.147 mM, MgSO_4_ 0.039 mM, KCl 0.134 mM, FeSO_4_/7H_2_O 0.7 μM) to inoculate wells of a 96 wells SensoPlate microplate (GREINER BIO-ONE) with 200 μL per well. Incubation was performed at 21°C, in the dark for 48 h prior to confocal acquisitions.

### Method details

#### Generation of fungal strains

The strains BcCHSIIIa::eGFP and eGFP^Cyt^ were obtained by *Agrobacterium tumefaciens-*mediated transformation of the *B. cinerea* strain B05.10.^54^ The BcCHSIIIa::eGFP strain was generated by the insertion, through homologous recombination, of an eGFP-tNiaD-NatR DNA fragment in frame with the BcCHSIIIa (Bcin04g03120) coding sequence (Figure S1A). This DNA fragment, containing eGFP optimized for *B. cinerea*,^43^ the terminator of the nitrate reductase from *B. cinerea* (tNiaD) and a nourseothricin resistance gene (NatR),^55^ was amplified from a donor plasmid. A 1031 bp fragment corresponding to the end of the BcCHSIIIa coding sequence, minus the STOP codon, was then amplified from B05.10 genomic DNA. A 924 bp fragment was amplified, corresponding to the 3′ UTR and terminator region of the BcCHSIIIa gene. The eGFP-tNiaD-NatR fragment was then assembled between the BcCHSIIIa coding sequence and the BcCHSIIIa terminator fragment using double-joint PCR.^56^ This DNA cassette was inserted by *In Vivo* Assembly (IVA) cloning^57^ into a *A. tumefaciens* transfer DNA carried by the p7 vector.^58^ Following verification by restriction enzyme digestion and sequencing, the vector was introduced in *A. tumefaciens* (strain LBA1126) and the transformed bacteria was used to mutagenize *B. cinerea*.^54^ The correct insertion of the cassette was tested using PCR with primers AtMT_S-IIIa-GFP-RFG-F (TCAGTTGGTTCATGTTATCTTCTTTCT) and AtMT_S_GFP-R (GGACATTGCACGGGATTACTT). Primer sequences used for plasmid construction are available from the authors upon request.

For the eGFP^Cyt^ strain, eGFP was placed under the control of the constitutive OliC promoter (pOliC) from *A. nidulans*^59^ and the tNiaD terminator. The pOliC-eGFP-tNiaD cassette was then inserted in the *A. tumefaciens* transfer DNA carried out by a pBHT2 plasmid containing a hygromycin resistance cassette.^60^ Transformation led to the obtention of *A. tumefaciens* transformants that were later used to transform conidia of the parental B05.10 strain. *B. cinerea* transformants displaying hygromycin and nourseothricin resistance where then screened using epifluorescence microscopy. One transformant with strong signal was then selected for use in this study. Primer sequences used for plasmid construction are available from the authors upon request.

#### Western blot analysis

Petri dishes containing PDA medium (THERMO SCIENTIFIC) were overlaid with cellophane and inoculated with 10 μL of conidia suspension (3 × 10^5^ conidia.mL^−1^ in MMII medium; see culture and growth conditions), using 15 dishes per strain (BcCHSIIIa::eGFP and parental B05.10). Cultures were incubated for 3 days in the dark at 21°C. Mycelia were harvested with cell scrapers, ground in liquid nitrogen, and resuspended in buffer containing SIGMA 7–9 50 mM, EDTA Na_2_ 10 mM, DTT 1 mM, sorbitol 1 M and NaCl 150 mM supplemented with a protease inhibitor cocktail (SIGMA, ref. P8215-5ml). Differential centrifugation was performed to enrich secretory vesicles: 1,000*g* for 10 min to pellet cellular debris, 36,000*g* centrifugation for 25 min to pellet large intracellular components, and 100,000*g* for 1 h to pellet intracellular vesicles. Pellets were resuspended in the same buffer and mixed with Laemmli (final concentrations: Tris 62.5 mM, SDS 2%, glycerol 10%) before incubation at 99°C for 3 min. Samples were loaded on Bolt Bis-Tris 4-12% gels (INVITROGEN) and run in Bolt MES SDS running buffer (INVITROGEN). Proteins were transferred to 0.2 μm nitrocellulose membranes (BIO-RAD), washed and blocked. Membranes were probed with anti-GFP from mouse IgG1κ primary antibodies (ROCHE, ref. 11 814 460 001) and HRP-conjugated goat anti-Mouse IgG (H + L) secondary Antibody HRP (INVITROGEN, ref. G-21040). Signal was detected using SuperSignal West Femto (Thermo Scientific ref. 34094) and imaged with a ChemiDoc XRS^+^ (BIO-RAD).

#### Image acquisition

Time-lapse acquisitions (10 min) were conducted using a laser scanning confocal microscope (ZEISS Observer 7 equipped with an LSM 800) and a Plan-Apochromat 63x/1.40 NA Oil DIC objective (ZEISS). Excitation was achieved using a 493 nm laser, and fluorescence emissions were detected within the range of 491–573 nm. The laser scanning was performed with an averaging of 16 on a single z-plane. To ensure a uniform acquisition speed, the size of the acquired region was standardized (295x224 pixels) leading to an acquisition rate of 4.455 s per frame. All acquisitions have a resolution of 7.3759 pixels per micron leading to a pixel size of 0.1356 × 0.1356 μm (0.0184 μm^2^ per pixel).

#### Tracking of apices

To compensate for the elongation of filaments occurring during the acquisition, a custom FIJI^61^ macro was designed to move a region of interest (ROI) of 50x50 pixels (6.78 × 6.78 μm) through time. Each frame was then cropped to obtain a 50x50 time-lapse acquisition with the apex (4 first micrometers of the hyphae) centered at each frame.

#### Region of apical fluorescence accumulation

To identify the region of apical fluorescence accumulation over time, time-projection was performed on each acquisition using the average intensity function in FIJI. We designed a FIJI macro to extract the top p% brightest pixels from each time-projection. The macro determines the corresponding threshold from the intensity histogram, retains those pixels as a mask, and measures the area, mean intensity, and roundness. To estimate the optimal p% threshold, area and roundness were quantified for thresholds ranging from 1% to 7% in steps of 0.5. The threshold needed to be small enough to remain focused on the region of fluorescence accumulation, yet large enough to exceed 0.5 μm^2^, as smaller regions are more affected by single-pixel differences that can disproportionately alter roundness estimates. Outputs at different p% values and for all acquisitions were analyzed in Python, and based on these comparisons a threshold of 4.5% was retained. This workflow was then applied consistently to all images to obtain the region of the 4.5% brightest pixels and the associated mean intensity, area, and roundness.

#### Morphological measures

The curvature of the apex was measured on non-cropped acquisitions using the Kappa plugin^62^ in FIJI. A 10 μm curved was placed to follow the apical dome, the curvature was then estimated as the curvature of the most apical point on the first frame of each acquisition. The hyphal width was calculated as the average of three measures performed in the distal part of the apical region (3.3 ± 1.2 μm) on the first frame of each non-cropped acquisition.

Kymographs were produced using the KymographBuilder^63^ plugin (v.3.0.0) in FIJI. For growth speed estimation, kymographs were produced using all time points of the acquisition (135 total points with a time between frames of 4.455 s).

#### From acquisition to time series

Before measuring fluorescence intensity, two approaches were used to suppress background pixels that would bias the measurements: one targeting extracellular pixels and one targeting cytosolic background signals.

For each acquisition, to limit the impact of extracellular pixels, a 50x50-pixel ROI was placed in the extracellular region. The mean fluorescence intensity in this ROI was measured for all time points and added to twice the associated standard deviation, so that 95% of extracellular pixels had values below the set threshold.^64^ All pixels with intensity values below this threshold were replaced by NaN. The threshold was computed independently for each acquisition. This approach restricts fluorescence measurements to the inside of the hyphae (termed threshold technique 1 in this section).

To limit the impact of cytosolic background fluorescence present in the acquisitions of the BcCHSIIIa::eGFP strain, a 16x16-pixel ROI was placed in the subapical region (5 ± 2 μm below the hyphal tip). The mean fluorescence intensity in this ROI was measured for the first 20 frames and added to twice the associated standard deviation, so that 95% of background signals were smaller than the threshold value.^64^ This threshold was used as an estimate of the cytosolic background intensity. All fluorescence values below this threshold were replaced by NaN. The threshold was computed independently for each acquisition. This approach restricts fluorescence measurements to regions of strong fluorescence (termed threshold technique 2 in this section).

Both approaches were applied independently to all acquisitions, each providing a distinct dataset. For each dataset, apices were tracked and cropped (see tracking of apices), and the mean fluorescence intensity was measured at all time points to produce time series (one per acquisition). For each time series the trend was identified using a Savitzky-Golay filter of window length 10 (polynomial degree of 2). Each signal was then subtracted by its own trend to obtain detrended signals. No normalization was applied to preserve the relative amplitude of short-term fluctuations.

#### Temporal Dynamics Clustering

Temporal Dynamics Clustering (TDC) is a feature-based clustering approach for time series.^44^ This approach was applied to detrended signals obtained after the threshold technique 1 (see from acquisition to time series). Following the approach detailed by Shuyang Li et al.,^44^ the absolute first-order difference was computed independently for all signals such as:

$$\Delta y_{t}=\left|y_{t}-y_{t-1}\right|$$

In our context, *Δy*_*t*_ corresponds to the absolute variation of fluorescence intensity between two successive frames. The second step of the TDC is the computation of *u* and of the subsequent *I*_*q*_, with

$$u=\frac{{\left(T-1\right)}^{2/3}}{\log\left(\log\left(T-1\right)\right)}$$

and

$$I_{q}=1-\frac{u}{T-1}$$

where T is the length of the original time series. In our study, the number of time points was consistent between acquisitions, leading to a single *u* and *I*_*q*_ values for all time series. *I*_*q*_ is a quantile index used to separate the elements of the first-order differences into a *main* section, containing all *Δy*_*t*_ values below or at the quantile *I*_*q*_, and a *tail* section containing all *Δy*_*t*_ values above *I*_*q*_. The *main* section thus contains moderate variations of fluorescence intensities, while the *tail* section contains extreme variation events. For each time-series three features were computed: the mean of elements in the *main* section (μ_main_), the standard deviation of elements in the main section (σ_main_), and the mean of elements in the *tail* section (μ_tail_). Each time series was therefore summarized by three metrics μ_main_, σ_main_ and μ_tail_ which characterize the variability of fluorescence in that time series. The resulting feature values (μ_main_, μ_tail_ and σ_main_) were used to cluster time series using *k*-means clustering.^59^ For numbers of clusters ranging from 2 to *N*-1 (where N is the number of time series), silhouette scores were computed to assess cluster quality, and the optimal number of clusters was defined as the configuration yielding the highest silhouette score with the minimal number of clusters.

#### Fast Fourier Transform

To characterize the temporal dynamics of fluorescence variations, Fast Fourier Transform (FFT) was used to analyze detrended time series obtained after the threshold technique 2 (see section from acquisition to time series). Time series were first smoothed using a Savitzky-Golay filter (window length 10, polynomial degree 2) in combination with a moving average filter (window size of 3). To improve frequency resolution, each time series was zero-padded to a total length of 16 384 time points.

For each time series *S*, the FFT of *S* (FFT(*S*)) was computed for all discrete frequency bins *f* using the fft function from NumPy.^65^ The amplitude *A*(*f*) associated with each frequency *f* was computed as

$$A\left(f\right)=2\frac{\sqrt{\text{Re}{\left[\text{FFT}\left(S\right)\left(f\right)\right]}^{2}+\text{Im}{\left[\text{FFT}\left(S\right)\left(f\right)\right]}^{2}}}{N}$$

where Re and Im denote the real and imaginary part of the FFT output, respectively, and *N* the number of points in the time series before zero padding. The factor 2 corrects the symmetric distribution of the FFT output over positive and negative frequencies.

For easier biological interpretation, amplitudes were plotted as a function of period (in seconds) instead of frequency (in Hz). To satisfy the Nyquist sampling theorem, periods shorter than 9 s were excluded. Additionally, only periods corresponding to at least four complete cycles within the 10-min time-lapse acquisitions were considered, excluding periods longer than 150 s.

#### Short-Time Fourier Transform analysis

To determine whether the periods detected by the Fast Fourier Transform were confined to specific portions of the time series, Short-Time Fourier Transform (STFT) analyses were performed on each signal using the scipy.signal.stft function in Python. The analysis used a Hann window with a segment length of 32 points and an overlap of 16 points (50%). Segments were then zero-padded to 16 384 points, consistent with the parameters used in the FFT analysis. Periods were calculated as the inverse of frequency, and only values corresponding to periods shorter than 150 s were retained. For each time segment, amplitudes were normalized by dividing all values by the maximum amplitude within that segment, yielding spectra scaled between 0 and 1 to facilitate comparison of peaks between fragments.

#### Sinusoidal model

For a period *p* and its associated amplitude *A*_*p*_, a sinusoidal model was generated using the equation

$$M\left(t\right)=A_{p}\cdot \sin\left(\frac{2\pi }{p}\left(t-\phi \right)\right)$$

where *φ* is the phase shift, defined as

$$\phi =\theta \frac{p}{360}$$

The phase (*φ*) was selected by minimizing the mean squared error between the model and the signal for *θ* between 0 and 360°, with an increment of 2°.

#### Control for z axis movement

Because the confocal time-lapse acquisitions were performed on a single z position, we tested whether temporal variations in fluorescence could originate from motion of the apical fluorescent region along the z axis, moving in and out of the acquired plane. Time-lapse z-stack imaging was performed with a laser scanning confocal microscope (ZEISS Observer 7 with LSM 800) using a Plan-Apochromat 63x/1.40 NA Oil DIC objective (ZEISS) after 48 h of incubation at 21°C in the dark in MMII (composition in section culture and growth conditions). The z-stack acquisition comprised 5 z-planes over 10 min with 1.806 s between planes. Of the five planes, four were analyzed (three intersecting the hypha and one extracellular), one plane that only partially intersected the hypha was excluded.

To measure the intensity of the cytosolic background fluorescence signal, a z-projection was performed by averaging pixel intensities across frames. The mean fluorescence intensity was then measured in a 16x16 ROI placed in the cytosolic region at all time points. A threshold was then computed as the mean plus twice the associated standard deviation. For all frames, and all z-planes, pixels below the threshold were replaced by NaN. Planes were then processed separately, the apex was tracked to a 50x50-pixel ROI, and mean fluorescence intensity was measured per frame to obtain time series.

Signals were detrended by subtracting a Savitzky–Golay trend (window length 10, polynomial degree 2), followed by a moving average (window length 3). Cross-correlation between pairs of signals was computed with numpy.correlate in full mode after mean-centering and standardizing each series by its standard deviation. Correlation sequences were normalized by dividing by the number of points. Lags were obtained with scipy.signal.correlation_lags, and for each pair the lag-0 correlation coefficient was extracted.

### Quantification and statistical analysis

#### Linear modeling and residual analysis

To assess linear relationships, we computed Pearson and Spearman correlations. Linear regression was performed using ordinary least squares (OLS) with the statsmodels package,^66^ adding a constant term to allow a non-zero intercept. Model fit was summarized by R^2^. The influence of individual observations on the OLS fit was assessed with Cook’s distance, using a threshold of 4/N to flag influential points.^67^ In addition, leave-one-out (LOO) analyses were performed to evaluate the robustness of the observed associations.

#### Statistics

Throughout the article, measurements are reported as mean ± standard deviation. For two-group comparisons, normality of each group was assessed with the Shapiro-Wilk test. When both groups did not deviate significantly from normality, homogeneity of variances was tested with Bartlett’s test, and either Student’s *t* test (for equal variances) or Welch’s *t* test (for unequal variances) were applied. If at least one group deviated from normality, we used the one-sided Mann-Whitney U test. In the main text and in legends, the test used and the associated *p*-value are reported, with the significance threshold set at 0.05. Sample sizes *n* corresponds to the number of hypha. Statistical analyses were performed in Python using the SciPy package.^68^ In plots, (ns) denotes non-significant tests (*p-*value > 0.05), (∗) indicates *p-*value < 0.05, (∗∗) indicates *p-*value < 0.01, and (∗∗∗) indicates *p-*value < 0.001. Given the modest sample sizes typical of long time-lapse live-cell imaging, these diagnostics were interpreted with caution.

All individual measurements per hypha used in the analyses are provided in Table S1.
