# Supplementary material for: Revisiting fungal polarized growth: from Spitzenkörper to crescent accumulation of secretory vesicles
Source: iScience. 2025 Nov 15;28(12):114067. doi: 10.1016/j.isci.2025.114067 (PMC12704382; doi:10.1016/j.isci.2025.114067)
Supplement: Document S1. Figures S1–S7 and Table S1 [file mmc1.pdf]

iScience, Volume *28*

Supplemental information

Revisiting fungal polarized growth: from Spitzenkörper to crescent accumulation of secretory vesicles

Adrien Hamandjian, Glen Calvar, Matthieu Blandenet, Mélanie Crumière, Nathalie Poussereau, Mathias Choquer, and Christophe Bruel

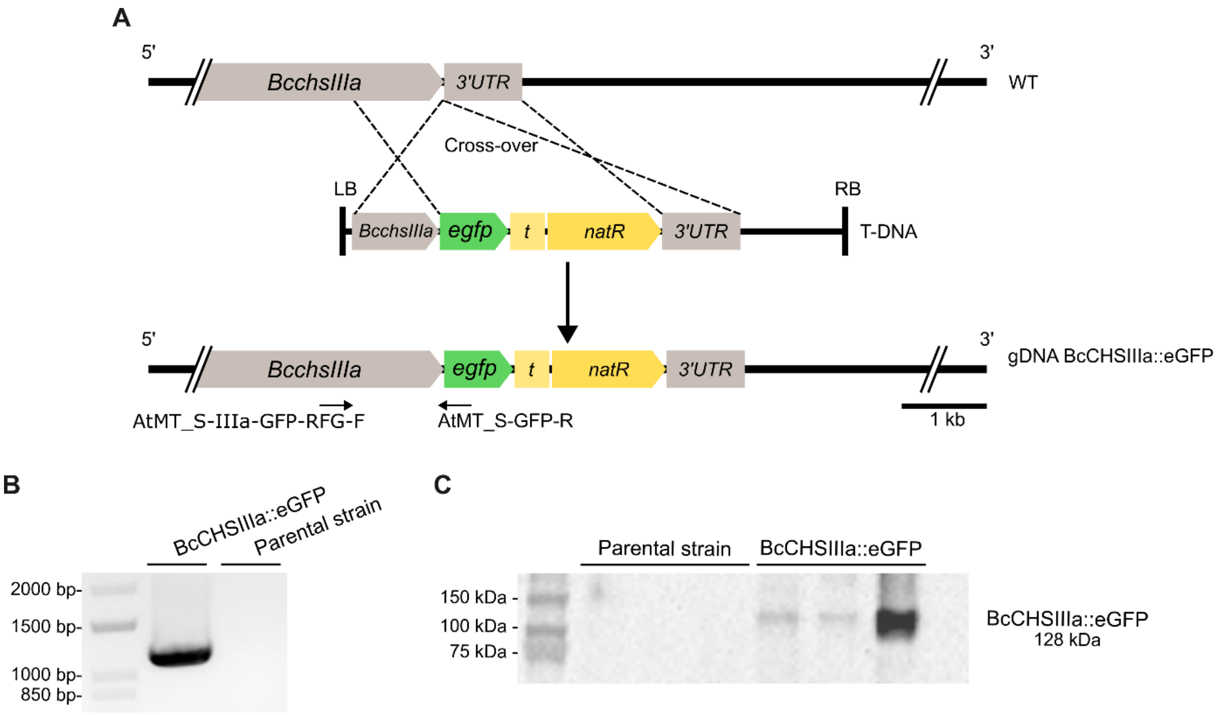

Figure S1. Construction of the BcCHSIIIa::eGFP strain, Related to STAR Methods

(A) Schematic representation of the insertion of the *BcchsIIIa::eGFP-nat^R-3'UTR* cassette by homologous recombination at the *BcchsIIIa* locus. A 1 031 bp fragment corresponding to the end of the *BcchsIIIa* coding sequence was fused with the coding sequence of the fluorescent protein eGFP, the *nat^R* resistance cassette (conferring resistance to nourseothricin), and the 3'UTR of *BcchsIIIa*. (B) PCR amplification using primers AtMT_S-IIIa-GFP-RFG-F and AtMT_S_GFP-R confirms the presence of the genetic construct in the BcCHSIIIa::eGFP strain, and its absence in the parental strain. (C) Western-blot detection of the chimeric BcCHSIIIa::eGFP protein in the BcCHSIIIa::eGFP strain. The three lanes correspond to fractions obtained by differential centrifugation at 1 000g (first lane), 36 000g (second lane), and 100 000g (third lane), used to enrich secretory vesicles.

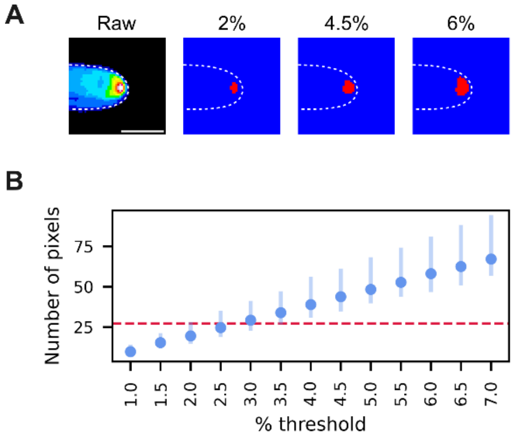

Figure S2. Selection of the intensity threshold used to define fluorescence accumulation regions, Related to Figure 1

(A) Examples of regions obtained by applying masks retaining the 2%, 4.5%, or 6% brightest pixels from the raw time projection. Scale bar, 3 µm. (B) Area of regions (in number of pixels) obtained for thresholds ranging from 1% to 7% with 0.5% steps (n = 13 hyphae). Dots represent mean area values and vertical bars indicate the minimum and maximum area. The horizontal line marks 0.5 μm^2 (27 pixels), below which roundness measurements may be biased by the small size of the region.

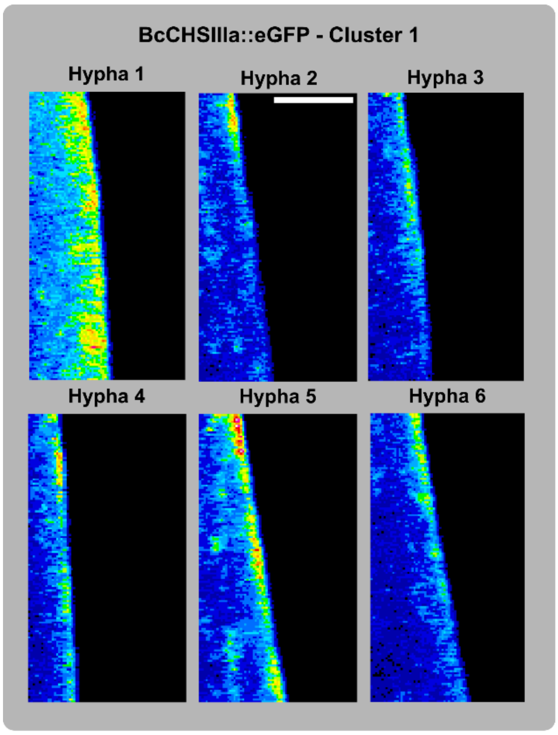

Acquisition	Growth rate ($\mu m.min^{-1}$)
Hypha 1	0.139
Hypha 2	0.233
Hypha 3	0.194
Hypha 4	0.104
Hypha 5	0.290
Hypha 6	0.305
Hypha 7	0.203
Hypha 8	0.151
Hypha 9	0.222
Hypha 10	0.399
Hypha 11	0.496
Hypha 12	0.422
Hypha 13	0.753
Hypha 14	0.836
Hypha 15	0.950
Hypha 16	0.304
Hypha 17	0.539
Hypha 18	0.477

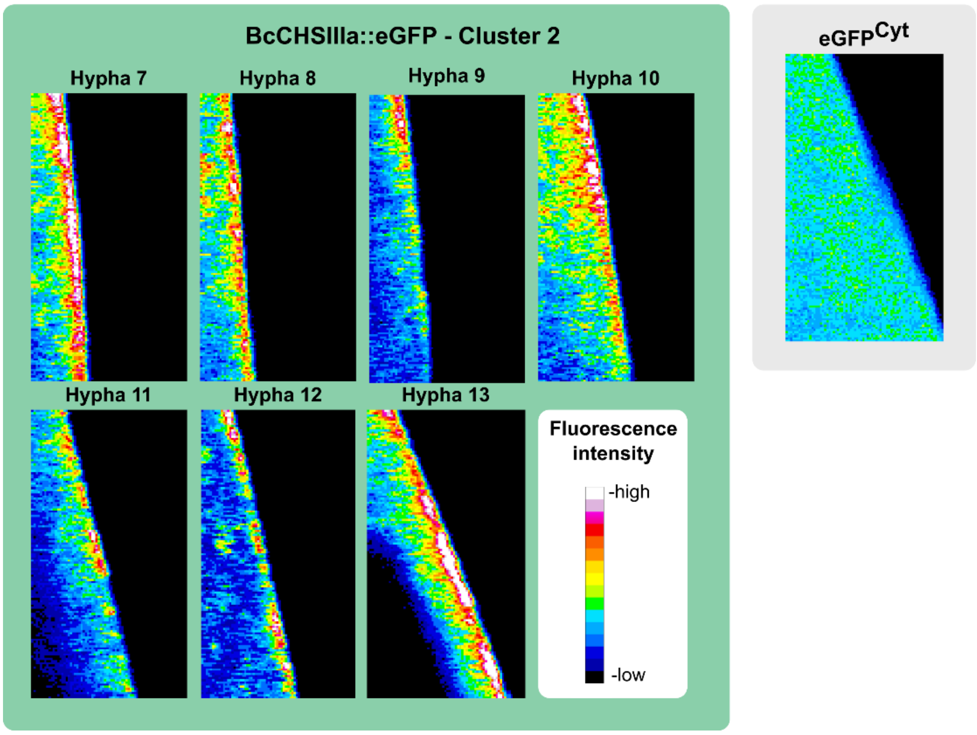

Figure S3. Kymographs obtained for all acquisitions of the BcCHSIIIa::eGFP strain, Related to Figure 1

Kymographs were generated along the elongation axis of individual hyphae to capture tip growth dynamics. For each acquisition, fluorescence intensity was measured along a 10 µm line at each time point corresponding to a total duration of 10 minutes (135 time points at 4.455-second intervals). In each kymograph, the x-axis represents the 10 µm line along the elongation axis, and the y-axis represents time over the 10-minute acquisition. Each kymograph corresponds to one hypha, with fluorescence

intensity color-coded as indicated. Scale bar, 5 µm. The table reports elongation rates obtained from kymographs for the 13 BcCHSIIIa::eGFP hyphae (Hyphae 1-13) and 5 eGFP^{Cyt} hyphae (Hyphae 14-18), in µm.min^{-1}. BcCHSIIIa::eGFP hyphae are grouped according to two distinct categories (Cluster 1 and Cluster 2) that were identified using the Temporal Dynamics Clustering (TDC) approach described in the main text.

A

Metric	Score	p-value
Shapiro-Wilk on growth rate measures	0,876	0,063
Shapiro-Wilk on roundness measures	0,978	0,965
Pearson correlation	0,769	0,002
Spearman correlatin	0,742	0,004
R^2	0,591	-
Maximal standardized residual	1,312	-
Maximal Cook's distance	0,235	-

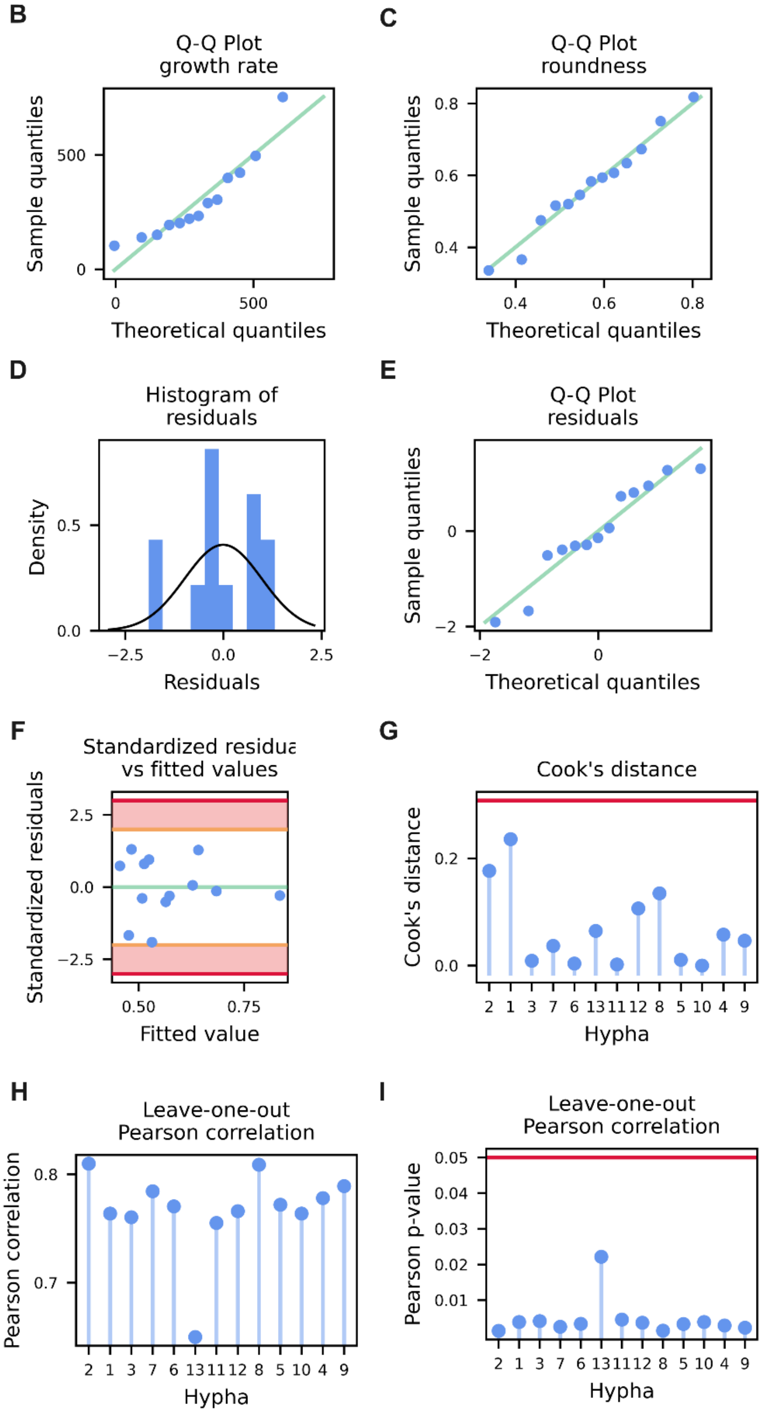

Figure S4. Validation metrics confirm linearity between roundness and growth rate, Related to Figure 1

(A) Summary of statistical metrics assessing the relationship between roundness and growth rate. (B, C) Q–Q plots for growth rate (B) and roundness (C). (D) Histogram of residuals from the linear model. (E) Q–Q plot of residuals. (F) Standardized residuals versus fitted values. Orange and red lines indicate thresholds at ± 2 and ± 3, commonly used to identify moderate and extreme outliers, respectively. (G) Cook's distance values were used to identify influential points. The red line marks the threshold of 0.308 (calculated as 4/n with n = 13). (H, I) Pearson correlation (H) and corresponding *p*-values (I) from the leave-one-out analysis. The x-axis indicates the hypha removed in each iteration. The red line in (I) corresponds to the significance threshold of 0.05.

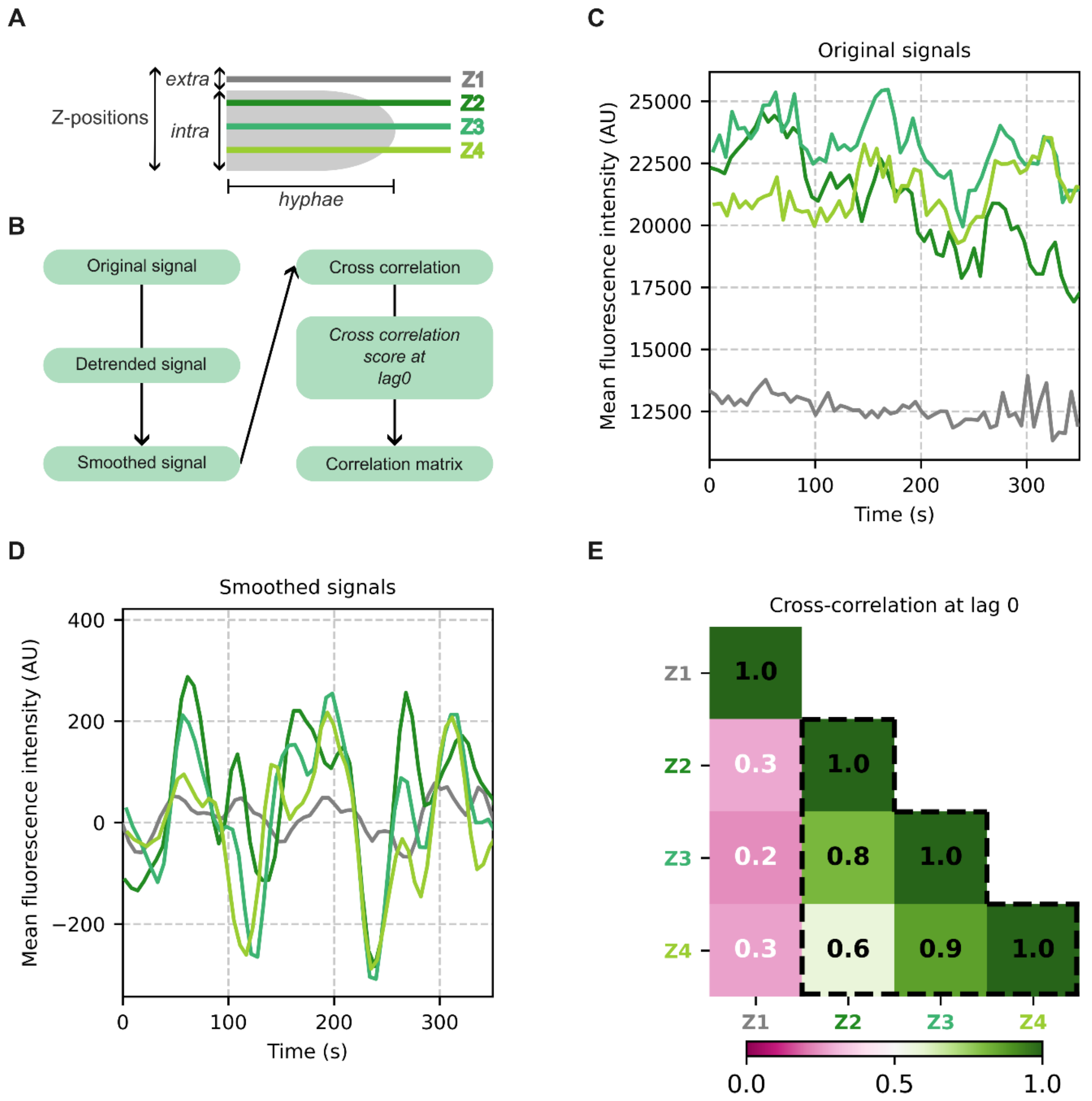

Figure S5. Cross-correlation analysis confirms consistent fluorescence oscillations across z-planes, Related to STAR Methods

(A) Schematic representation of a hypha and the four focal planes acquired in a time-lapse z-stack (1.806 s between z-planes). Z1 corresponds to an extracellular position, whereas Z2-Z4 intersect the fungal cell. (B) Overview of the signal-processing pipeline. (C) Original signals corresponding to the mean fluorescence intensity at the hyphal tip in planes Z1-Z4. (D) Processed (detrended and smoothed) fluorescence signals. (E) Cross-correlation matrix at lag-0 between processed signals. Time projection was performed and the roundness of the region of fluorescence accumulation measured in Z2-Z4 with roundness values of 0.766, 0.744 and 0.731, respectively. Correlation analysis showed low similarity between the extracellular plane Z1 and the intracellular planes (correlation ≤ 0.3), whereas intracellular planes displayed strong to very strong correlations with one another (correlation = 0.6-0.9). These results indicate that signals from Z2-Z4 reflect a single, consistent intracellular fluorescence source. Periodic fluorescence variations therefore originate from changes in apical fluorescence accumulation rather than from displacement along the z-axis.

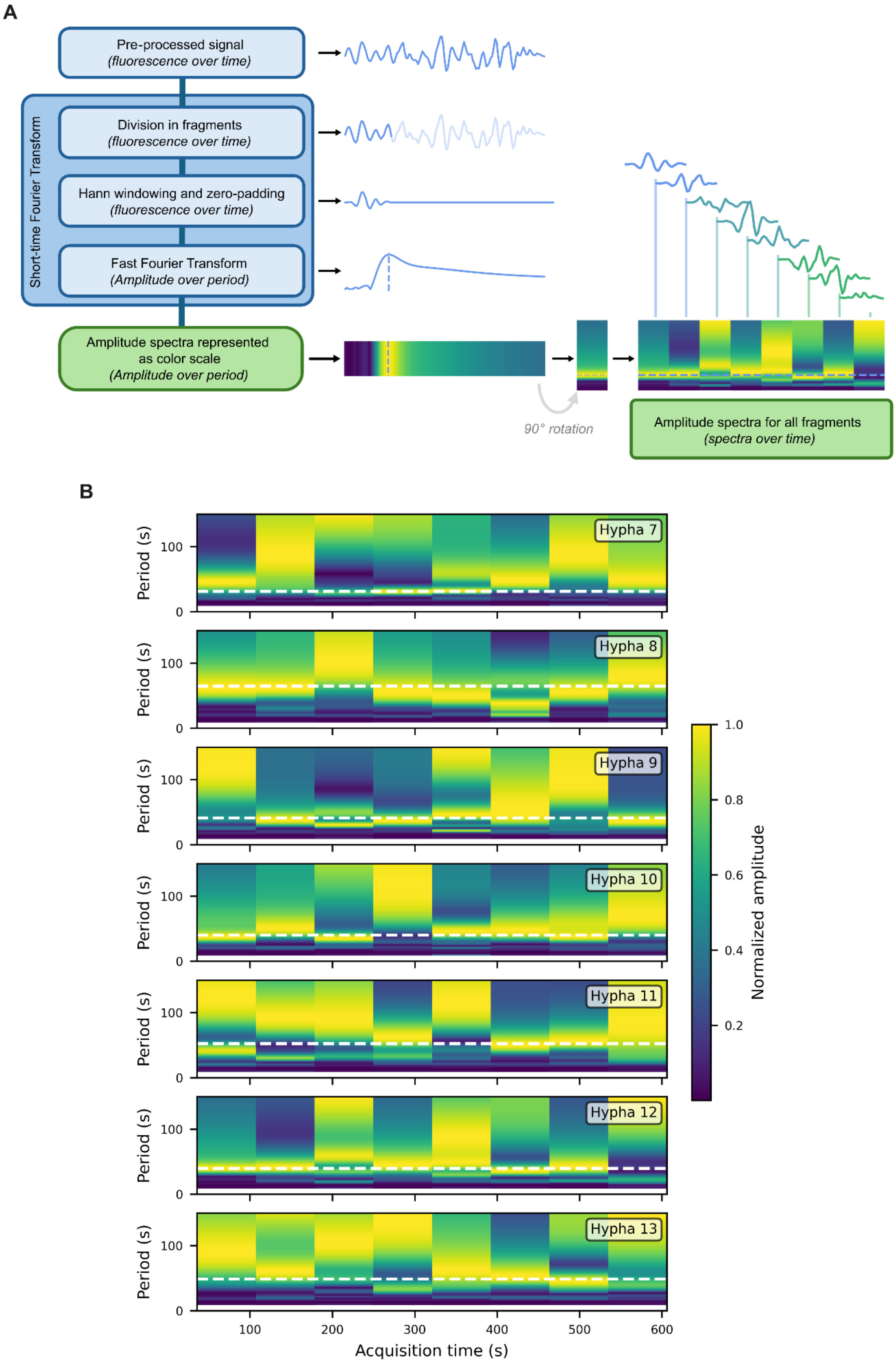

Figure S6. Short-time Fourier transform (STFT) reveals temporal stability of fluorescence oscillations in Cluster 2 acquisitions, Related to STAR Methods

(A) Overview of the STFT pipeline. Pre-processed fluorescence signals were divided into overlapping fragments, Hann-windowed to limit edge effects, zero-padded, and analyzed using the Fast Fourier Transform (FFT). Amplitude spectra obtained from the FFT were then represented as color-coded maps and concatenated across fragments to produce spectra over time. (B) Time-resolved amplitude spectra for acquisitions of hyphae 7-13, all belonging to Cluster 2. The y-axis indicates period, and the x-axis indicates acquisition time. Color represents normalized amplitude. The white dashed line marks the dominant oscillation period identified for each hypha.

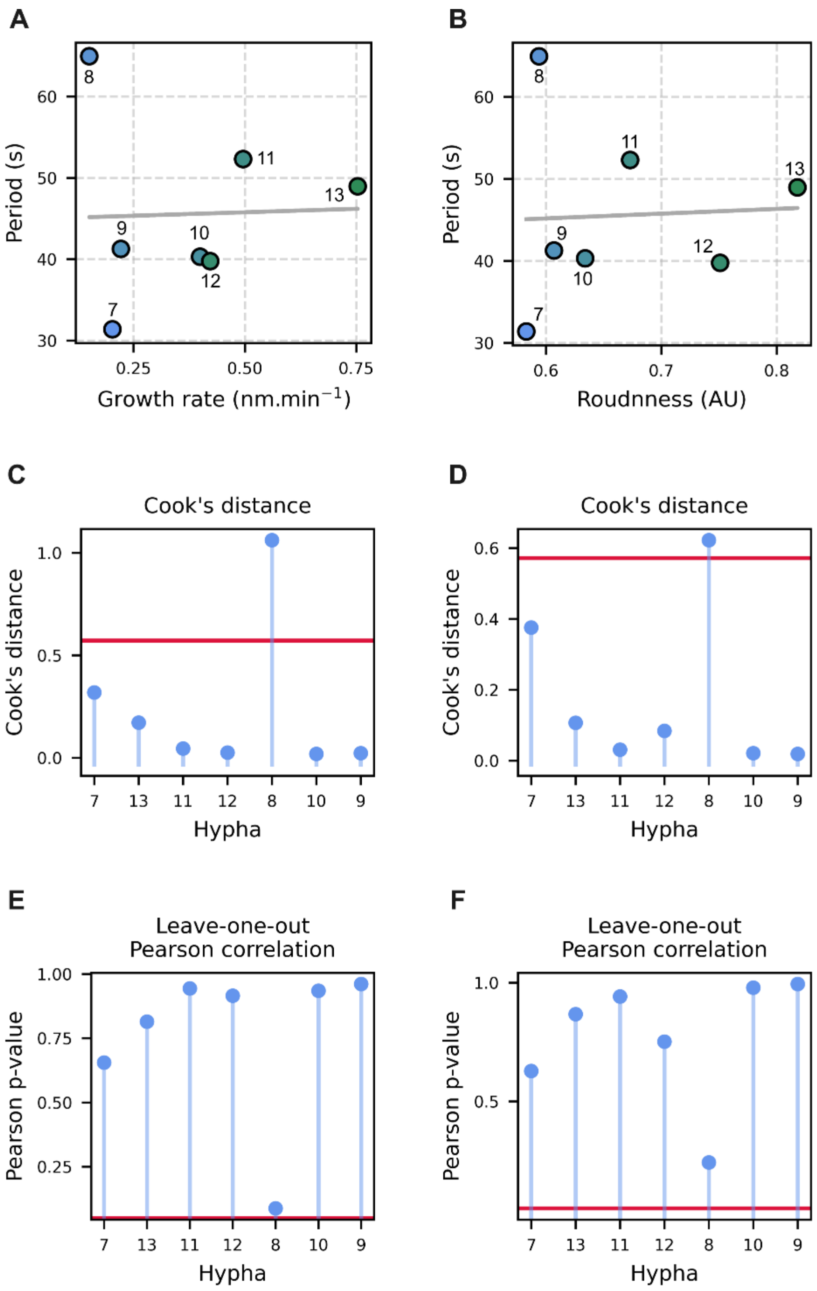

Figure S7. Periods of fluorescence variation are not linearly correlated with growth rate or the roundness, Related to Results *Fluorescence variations are periodic in hyphae exhibiting a Spitzenkörper*

(A) Scatter plot of oscillation period versus hyphal elongation rate for acquisitions in Cluster 2. (B) Scatter plot of oscillation period versus roundness of the fluorescence accumulation region in the same hyphae. Points in (A) and (B) are numbered to match the corresponding hyphae shown in Figure 1C. (C, D) Cook's distance values for the relationship between period and growth rate (C) and between period and roundness (D). Red horizontal lines mark the threshold for influential observations computed as 4/n with n = 7. (E, F) Leave-one-out Pearson correlation analyses for the relationship in (A) and (B). Red horizontal lines indicate the significance threshold *p*-value 0.05.

		Morphological and growth descriptors				Temporal Dynamics Clustering				Region of fluorescence accumulation		Fourier Transform Analysis
Acquisition	Strain	Growth rate ($\mu m.min^{-1}$)	Hyphal width (µm)	Max curvature	Median fluoresce-nce signal	TDC Cluster	μ_{main}	μ_{tail}	σ_{main}	Area (μm^2)	Roundness	Period (s)
Hypha 1	BcCHSIIIa::eGFP	0.139	5.197	0.828	21179	1	218.0	694.5	129.8	0.956	0.336	
Hypha 2	BcCHSIIIa::eGFP	0.233	3.187	0.989	8553	1	153.6	569.4	106.2	1.084	0.366	
Hypha 3	BcCHSIIIa::eGFP	0.194	3.058	1.339	8844	1	179.1	498.8	102.8	0.698	0.475	
Hypha 4	BcCHSIIIa::eGFP	0.104	2.320	1.447	8237	1	184.9	599.3	127.9	0.662	0.516	
Hypha 5	BcCHSIIIa::eGFP	0.290	2.305	1.820	11753	1	186.9	532.4	122.3	0.735	0.520	
Hypha 6	BcCHSIIIa::eGFP	0.305	3.357	1.401	8401	1	146.9	410.4	81.0	0.956	0.546	
Hypha 7	BcCHSIIIa::eGFP	0.203	2.413	1.799	20606	2	398.4	1260.9	271.3	0.680	0.583	31.89
Hypha 8	BcCHSIIIa::eGFP	0.151	2.378	1.971	17419	2	330.0	963.3	222.9	0.698	0.594	64.93
Hypha 9	BcCHSIIIa::eGFP	0.221	2.152	1.807	10976	2	328.7	910.2	199.4	0.680	0.607	41.26
Hypha 10	BcCHSIIIa::eGFP	0.399	2.943	1.246	20594	2	421.5	1241.9	278.2	0.809	0.634	40.30
Hypha 11	BcCHSIIIa::eGFP	0.496	2.334	1.969	12248	2	375.0	1200.5	237.2	0.717	0.673	52.29
Hypha 12	BcCHSIIIa::eGFP	0.422	3.088	1.670	13031	2	333.0	1057.3	231.3	0.698	0.751	39.75
Hypha 13	BcCHSIIIa::eGFP	0.753	3.316	1.190	17766	2	381.3	1199.2	234.0	1.103	0.818	48.95
Hypha 14	$eGFP^{Cyt}$	0.836	2.485	1.606	17022	1	143.3	506.9	95.9			
Hypha 15	$eGFP^{Cyt}$	0.095	2.311	2.202	20495	1	159.2	515.8	105.2			
Hypha 16	$eGFP^{Cyt}$	0.304	2.702	1.720	24199	1	214.2	612.4	129.0			
Hypha 17	$eGFP^{Cyt}$	0.539	4.281	1.229	31244	1	154.4	433.4	85.2			
Hypha 18	$eGFP^{Cyt}$	0.477	2.858	1.252	36624	1	245.7	605.9	135.7			

Table S1. Summary of key quantitative metrics measured per hypha
